# Numerical simulation to optimize power conversion efficiency of an FTO/GO/Cs_2_AgBiBr_6_/Cu_2_O solar cell

**DOI:** 10.1039/d4ra01559b

**Published:** 2024-06-13

**Authors:** Ghulam M. Mustafa, Bisma Younas, Sadaf Saba, Zainab Mufarreh Elqahtani, Norah Alwadai, Sikandar Aftab

**Affiliations:** a Department of Physics, Division of Science and Technology, University of Education Lahore Punjab 54770 Pakistan dr.ghulam.muhammad@ue.edu.pk; b Department of Physics, University of Lahore Lahore 53700 Pakistan; c Center of Excellence in Solid State Physics, University of the Punjab Lahore Pakistan; d Department of Physics, College of Sciences, Princess Nourah bint Abdulrahman University P.O. Box 84428 Riyadh 11671 Saudi Arabia; e Department of Intelligent Mechatronics Engineering, Sejong University 209 Neungdong-ro, Gwangjin-gu Seoul 05006 South Korea

## Abstract

Efficient conversion of solar power to electrical power through the development of smart, reliable, and environmentally friendly materials is a key focus for the next-generation renewable energy sector. The involvement of degradable and toxic elements present in hybrid perovskites presents serious concerns regarding the commercial viability of these materials for the solar cell industry. In this study, a solar cell with a stable, nondegradable, and lead-free halide-based double perovskite Cs_2_AgBiBr_6_ as the absorber layer, Cu_2_O as a hole transport layer, and GO as the electron transport layer has been simulated using SCAPS 1D. The thickness of the absorber, electron transport, and hole transport layers are tuned to optimize the performance of the designed solar cell. Notably, perovskite solar cells functioned most efficiently with an electron affinity value of 4.0 eV for Cu_2_O. In addition, the effect of variation of series resistance and temperature on generation and recombination rates, current density, and quantum efficiency has been elaborated in detail. The findings of this study provide valuable insight and encouragement toward the realization of a non-toxic, inorganic perovskite solar device and will be a significant step forward in addressing environmental concerns associated with perovskite solar cell technology.

## Abstract 2

Efficient conversion of solar power to electrical power through the development of smart, reliable, and environmentally friendly materials is a key focus for the next-generation renewable energy sector.

## Graphical abstract

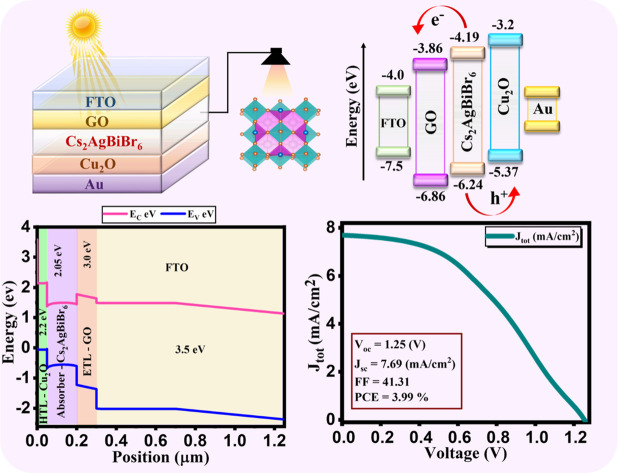

## 1. Introduction

In the 21st century, humanity stands at the precipice of an unprecedented energy crisis, confronting a global challenge that transcends borders, economies, and ideologies. The rapid depletion of finite fossil fuel reserves, coupled with escalating environmental concerns, has spurred a global quest for innovative and efficient renewable energy sources.^1^ Among these, solar energy emerges as a preeminent contender, offering a tantalizing solution to our energy woes. Solar cells, the vanguard of solar energy conversion technology, encapsulate the audacious promise of harnessing the sun's inexhaustible energy reservoir.^2^ Through the marvels of semiconductor physics, these photovoltaic devices transmute photons into electricity, providing a clean and boundless power source.^3^ Over the decades, relentless research and technological advancements have propelled solar cells from nascent prototypes to commercially viable energy generators. Today, they adorn rooftops, power remote communities, and even propel spacecraft into the cosmic expanse, a testament to their transformative potential.^4^

In this context, perovskite-based solar cells have developed as a groundbreaking innovation in photovoltaic technology. The versatility of perovskite materials allows for low-cost and higher-efficiency solar cell production through relatively simple manufacturing processes, making them a formidable contender in the renewable energy arena.^5^ With the prospective to surpass the efficiency of traditional Si-based solar cells (26.7%), perovskite-based technologies promise to democratize access to clean energy, rendering it more affordable and accessible to diverse populations worldwide.^6^ In current ages, perovskite solar cells (PSCs) have garnered noteworthy research interest as an innovative methodology in solar photovoltaic technology, primarily owing to their notable improvement in power conversion efficiency (PCE), which has surged from 3.8% to 32.72% till 2022.^7^ The general formulation of perovskite compounds is ABX_3_, here A represents a cation, typically a larger organic or inorganic ion, B represents another cation, usually a smaller metallic ion and X represents an anion, often a halide such as iodide (I), bromide (Br), or chloride (Cl). From the perspective of perovskite solar cells, the most commonly used material is a hybrid organic-inorganic perovskite, which involves an organic cation like formamidinium (HC(NH_2_)^2+^) or methylammonium (CH_3_NH^3+^), and an inorganic cation like lead (Pb^2+^).^8^ This leads to the general formula CH_3_NH_3_PbX_3_, where X might be I, Br, or Cl. Among these, the most widely studied and utilized perovskite material for solar cell formation is methylammonium lead triiodide (CH_3_NH_3_PbI_3_).^9^ This compound has shown exceptional photovoltaic properties, including high light absorption, long carrier lifetimes, and good charge carrier mobility.^10^ These characteristics contribute to its high-power conversion efficiency, crafting it as a leading candidate for commercial solar cell device applications. However, it's worth noting that the use of Pb in perovskite solar cells has raised environmental and health concerns.^11^ Due to an escalating demand for environmentally safe, lead-free perovskite solar cells, scientists have embarked on a quest to pioneer a range of lead-free perovskite materials. Researchers are actively exploring lead-free alternatives to address these issues while maintaining high-efficiency levels.^12,13^ This endeavor involves substituting Pb^2+^ with non-toxic counterparts, most notably bivalent elements like Ge^2+^ and Sn^2+^, in contrast to conventional lead-based perovskites. Yet, it's crucial to note that Ge^2+^ and Sn^2+^ in lead-free PSCs present a vulnerability to oxidation, thus exhibiting diminished stability.^14^ To circumvent this challenge, an innovative approach has emerged wherein Pb^2+^ is replaced by hetero-valent M^3+^ ions, particularly Bi^3+^. Bi^3+^ stands out for its non-toxic nature, its iso-electronic resemblance to Pb^2+^, and its ability to form stable semiconducting halides.^15^ Bi-centered perovskite solar cells showcase enhanced charge carriers' diffusion capabilities attributed to their inherently low trap density and reduced defect states. Nevertheless, it's worth mentioning that the introduction of high-charged bismuth (Bi^3+^) ions into 3D A^1+^M^2+^X_3_ crystal structure leads to a notable decline in optoelectronic performance when compared to their lead-based counterparts. In a strategic move to address these shortcomings, the elpasolite structure, also identified as a double perovskite crystal structure, has emerged as a promising solution.^16,17^ This entails the inclusion of a Bi^3+^ anion, fundamentally altering the structural composition. The elpasolite structure adheres to the general formula A_2_M^1+^M^3+^X_6_, wherein A, X, M^1+^, and M^3+^ correspond to monovalent cations, halide anions (Br^−^, Cl^−^, I^−^), inorganic cations (Ag^+^, Cu^+^, Rb^+^, Au^+^, Na^+^, K^+^ and In^+^), and either organic/inorganic cations (Bi^3+^ or Sb^3+^), respectively. Furthermore, some promising lead-free perovskite materials include tin-based perovskites (*e.g.*, Sn-based) and double perovskites (*e.g.*, Cs_2_AgBiBr_6_). Though, these materials show potential, still they undergoing extensive research and development to achieve the efficiency levels observed with Pb-based perovskites. Additionally, Cs_2_AgBiBr_6_ exhibits a stable crystal structure, a vital characteristic for ensuring the long-term performance and reliability of solar cells. This stability not only enhances the material's efficiency but also extends its operational lifespan, making it a viable candidate for widespread solar energy deployment. Furthermore, the tunable band gap of Cs_2_AgBiBr_6_ offers a versatile platform for optimizing its light-absorbing properties, paving the way for tailored solar cell designs optimized for specific environmentally friendly conditions and energy conversion efficiency. In essence, Cs_2_AgBiBr_6_ represents a significant advancement in the pursuit of cleaner, safer, and more sustainable solar energy technologies.^18^

Consequently, the research endeavors in this domain are advancing at an astonishing rate, with a multitude of studies focused on exploring novel perovskite materials. In the study of Ahmed *et al.*, they examined the enhancement of performance of CH_3_NH_3_Pb(I_1−*x*_Br_*x*_)_3_-based perovskite solar cells by investigating various electron transport layer (ETL) materials. Through conduction band offset, the researchers aimed to optimize the device's efficiency. Their findings shed light on the crucial role of ETL materials in enhancing the efficiency of PSCs, offering valuable insights for further advancements in this promising renewable energy technology.^19^ In the study of Alias *et al.*, the influence of aluminum (Al) incorporation into the ZnO electron transport layer (ETL) has been examined in PSCs. This research focused on the effects of Al on the performance of the ETL. Through their investigation, the authors aimed to improve the stability and efficiency of PSCs. The findings revealed the potential benefits of incorporating Al into the ZnO layer, offering valuable insights for the advancement of perovskite-based photovoltaic technology. This work represents a significant step towards optimizing electron transport layers for more efficient solar cell devices.^20^ Rai *et al.* conducted a study on the optimization of the hole transporting layer (HTL) for improving the performance of Pb-free double PSCs. Utilizing numerical simulations, the researchers aimed to identify the most effective HTL material and its thickness. Their findings revealed that careful selection of the HTL significantly improved the cell's performance. The report gives important visions for the expansion of environmentally friendly and efficient solar cell methodologies.^21^ Das *et al.* conducted a study focused on optimizing HTL material for enhancing the efficiency of Pb-free double perovskite Cs_2_AgBiBr_6_ solar cells. Through numerical simulations, the researchers aimed to identify the most effective HTL material. Their findings emphasized the crucial role of proper HTL selection in improving the cell's performance. This study provides a significant understanding of the progress of effective and eco-friendly solar cell technologies.^22^ Alam *et al.* conducted a numerical simulation of Cs_2_AgBiBr_6_ PSCs, employing ZnO nanorods (ZnO-NR) as the ETL and CuI as the HTL. Their study aimed to evaluate the performance of this configuration. The simulation results demonstrated promising efficiency and stability, showcasing the potential of Cs_2_AgBiBr_6_ perovskite in solar cell technology. This research offers valuable insights for the advancement of effective and sustainable photovoltaic device applications.^23^

**Fig. 1:**
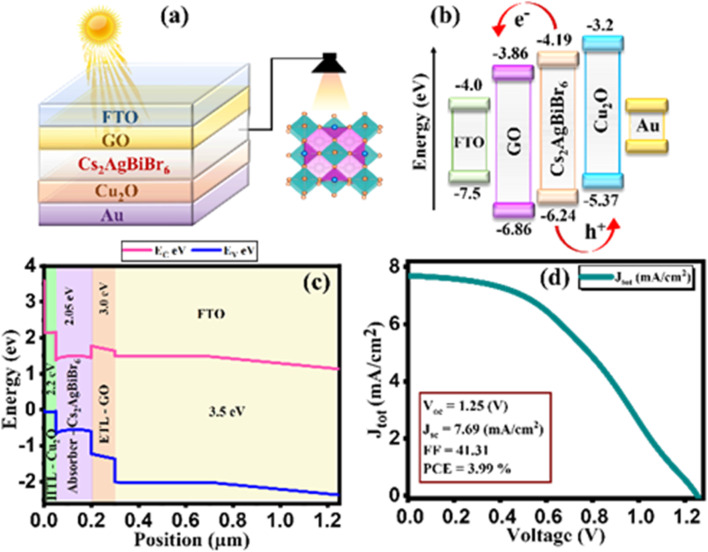
(a) Device configuration of simulated perovskite solar cell, (b) energy-band diagram without contact between layers (c) energy-band diagram after contacts between layers (d) current density–voltage curve.

This study delves into the efficiency of Pb-free Cs_2_AgBiBr_6_ halide perovskite solar cells (PSCs) using 1D solar cell capacitance simulator (SCAPS-1D) software. The investigation encompasses the employment of graphene oxide (GO) as an ETL and copper oxide (Cu_2_O) as an HTL, with gold (Au) serving as the back contact metal. The hetero-structure design of FTO/GO/Cs_2_AgBiBr_6_/Cu_2_O/Au for the device, band diagram, grading of energy parameters, and *J*–*V* plot is demonstrated in Fig. 1(a–d). Moreover, the ultimate model incorporates an absorber layer with a thickness of 1500 nm and a defect density of 3.678 × 10^16^ cm^−3^. Additionally, doping levels for HTL and ETL are established at 9 × 10^15^ and 1 × 10^15^ cm^−3^. As illustrated in Fig. 1, it showcases distinctive characteristics of perovskite solar cells, specifically depicting the final *J*–*V* plot. Notably, open circuit voltage (*V*_oc_) is measured as 1.25 V, while short circuit current density (*J*_sc_) is reached at 7.69 mA cm^−2^. Furthermore, the value of the Fill Factor (FF) is recorded as 41.31%, leading to the power conversion efficiency (PCE) of 3.99% as cited in Table 1.^24^ Additionally, an exploration into the influence of ETL and HTL layers performance, with absorber, HTL, and ETL thickness, series resistance, operational temperature, and quantum efficiency (QE) response was conducted.

**Table 1:** Our simulation results of current density–voltage curves, for FTO/GO/Cs_2_AgBiBr_6_/Cu_2_O/Au cell

FTO/GO/Cs_2_AgBiBr_6_/Cu_2_O			
*V*_OC_ (V)	*J*_SC_ (mA cm^−2^)	FF (%)	PCE (%)
1.25	7.69	41.31	3.99

## 2. Simulation methodology

**Fig. 2:**
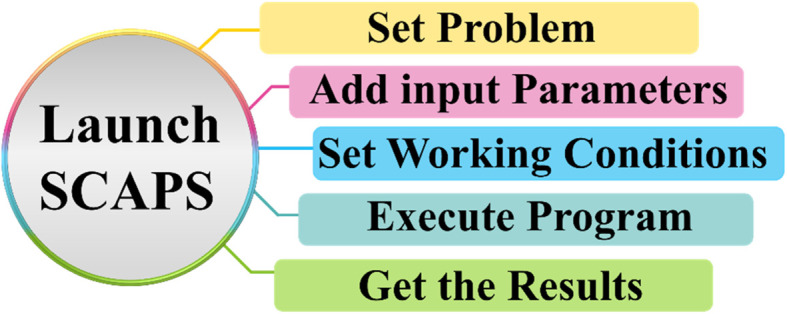
SCAPS simulation procedure.

The simulation of solar cells using Solar Cell Capacitance Simulator (SCAPS 1-D) required various input parameters including the thickness of involving layers, bandgap, dielectric constant, electron affinity, carrier density, temperature, *etc.*, and all these parameters are enlisted in Table 2. These input parameters have a direct influence on simulation outcomes. Key factors like material parameters, device geometry, and operational conditions directly impact the accuracy and relevance of simulated results such as carrier transport, recombination rates, and electrical characteristics. The sequence and summary of the simulation method are presented in Fig. 2. This software was developed at the Department of Electronics and Information Systems at the University of Gent, Belgium.^25^ This software is written in C programming language and allows for the calculation of various device architecture parameters, including grading, generation and recombination rates, and defects. Previous studies have already utilized SCAPS 1-D for simulating PSCs.^26^ This software employs numerical methods to solve Poisson's and continuity equations, enabling the calculation of parameters such as open-circuit voltage (*V*_OC_), photogenerated current density (*J*_SC_), fill factor (FF), and PCE.^27,28^ Below are the expressions for Poisson's equation (eqn (1)), electron continuity equation (eqn (2)) and hole continuity equation (eqn (3)):^29^1
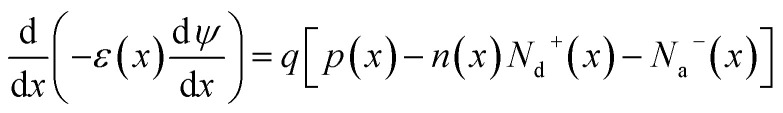
2
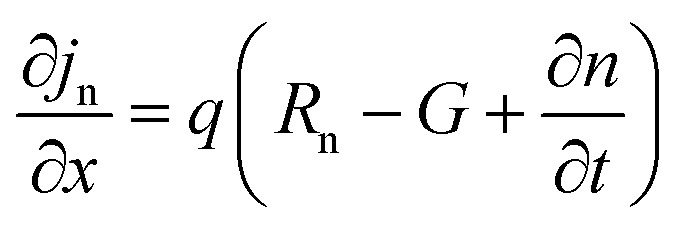
3
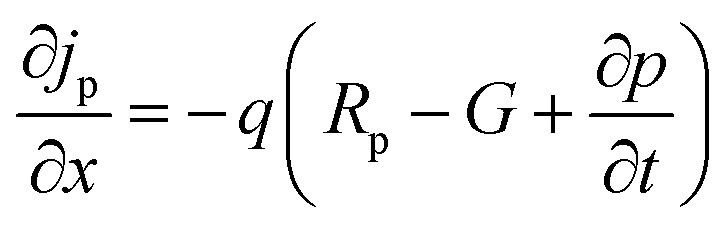
Here, *ε* represents permittivity, *q* signifies the charge of an electron, *ψ* symbolizes electrostatic potential, *n* denotes overall electron density, *p* stands for total hole density, *N*_d_^+^ represents the concentration of ionized donor as doping, *N*_a_^−^ indicates the concentration of ionized acceptor as doping. Additionally, *j*_n_ and *j*_p_ are utilized to denote electron and hole current densities, while *R*_n_ and *R*_p_ correspond to net recombination rates for electrons and holes/unit volume, respectively. Finally, *G* represents generation rate/unit volume.

**Table 2:** Physical parameters of the PSC used in the simulation study

Parameters	Cu_2_O (HTL)	Cs_2_AgBiBr_6_	GO (ETL)	FTO
Thickness (nm)	100	150	100	400
Bandgap (eV)	2.17	2.05	3.0	3.5
Electron affinity (eV)	3.2	4.19	3.86	4.0
Dielectric permittivity	7.1	5.8	9.0	9.0
CB effective density of state (cm^−3^)	2.5 × 10^18^	1.000 × 10^16^	2.2 × 10^18^	2.2 × 10^17^
VB effective density of state (cm^−3^)	2.5 × 10^18^	1.000 × 10^16^	1.8 × 10^19^	1.8 × 10^17^
Electron thermal velocity (cm s^−1^)	1.000 × 10^7^	1.000 × 10^7^	1.000 × 10^7^	1.000 × 10^7^
Electron thermal velocity (cm s^−1^)	1.000 × 10^7^	1.000 × 10^7^	1.000 × 10^7^	1.000 × 10^7^
Electron mobility (cm^2^ V^−1^ s^−1^)	2.0 × 10^2^	11.81	1.0 × 10^2^	2.0 × 10^1^
Hole mobility (cm^2^ V^−1^ s^−1^)	8.0 × 10^2^	0.49	3.0 × 10^1^	1.0 × 10^1^
Shallow uniform donor density *N*_D_ (cm^−3^)	0	1.000 × 10^19^	1 × 10^15^	2.0 × 10^17^
Shallow uniform acceptor density *N*_A_ (cm^−3^)	9.0 × 10^15^	1.000 × 10^19^	0	0
Total density (*N*_t_)	1.0 × 10^14^	3.678 × 10^16^	1.0 × 10^14^	1.0 × 10^15^
Ref.	30	31	30	30

## 3. Results and discussion

### 3.1. Effect of thickness of hole and electron transport layer

**Fig. 3:**
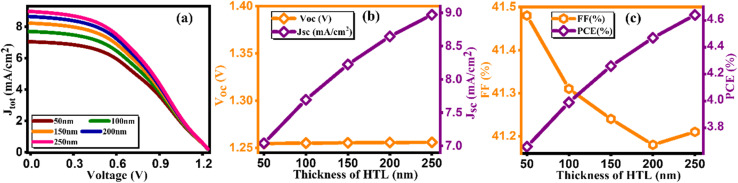
Variation of (a) current density *versus* voltage, (b) *V*_oc_ and *J*_sc_, and (c) FF and PCE with thicknesses of HTL.

During this simulation, we tuned the thickness of the hole and electron transport layer to optimize the performance of solar cells.^32^ The optimization of transport layer thickness stands as a crucial endeavor in enhancing the efficiency of PSCs. In this pursuit, we finely tuned the thickness of the hole transport layer in the range of 0–200 nm and plotted the current density *versus* voltage graph at different thicknesses as depicted in Fig. 3(a). Furthermore, the values of *J*_SC_, and PCE exhibit discernible increments in response to varying hole transport layer thicknesses as tabulated in Table 3. This suggests that a thicker HTL enhances charge transport and collection within the device, leading to higher current generation and overall device performance. However, there is a decrease in open-circuit voltage (*V*_OC_) and fill factor (FF) as the HTL thickness increases, which could be attributed to increased series resistance and recombination losses within the device.^33^

**Table 3:** Variation of performance indicators with thickness of HTL

Thickness (nm)	*V*_OC_ (V)	*J*_SC_ (mA cm^−2^)	FF (%)	PCE (%)
0	1.35	6.24	41.71	3.54
50	1.25	7.04	41.48	3.66
100	1.25	7.69	41.31	3.99
150	1.25	8.22	41.24	4.26
200	1.25	8.64	41.18	4.47

**Fig. 4:**
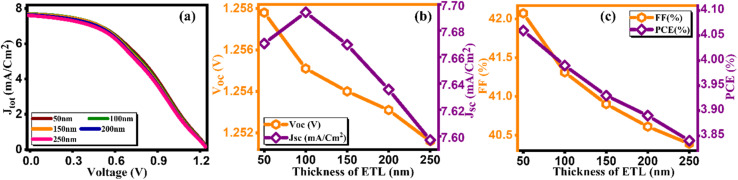
Variation of (a) current density *versus* voltage, (b) *V*_oc_ and *J*_sc_, and (c) FF and PCE with thicknesses of ETL.

Notably, the paramount parameter, power conversion efficiency (PCE) (Fig. 3(c)), demonstrates a noteworthy increase, achieving a peak of 4.47% at an optimal thickness of 200 nm.^34^ Conversely, in tuning the thickness of the electron transport layer (ETL), the *J*–*V* curve along with other key factors are presented in Fig. 4(a–c) and computed key parameters, including *V*_OC_, *J*_SC_, FF, and PCE, are exhibited in Table 4. It is clearly seen that the increase in the thickness of the ETL leads to the degradation in the PV parameters for ETL, thereby leading to a decrement in PCE. This is due to the inefficient transport of charge carriers to the electrodes, the increase in series resistance that degrades the FF, and the increase in the probability of recombination with increasing ETL thickness. The same kind of behavior is reported by Hossain *et al.*^35^

**Table 4:** Variation of performance indicators with thickness of ETL

Thickness (nm)	*V*_OC_ (V)	*J*_SC_ (mA cm^−2^)	FF (%)	PCE (%)
0	1.26	7.62	43.18	4.15
50	1.25	7.67	42.07	4.06
100	1.25	7.69	41.31	3.99
150	1.25	7.66	40.9	3.93
200	1.25	7.63	40.61	3.89

### 3.2. Effect of thickness of absorber layer

**Fig. 5:**
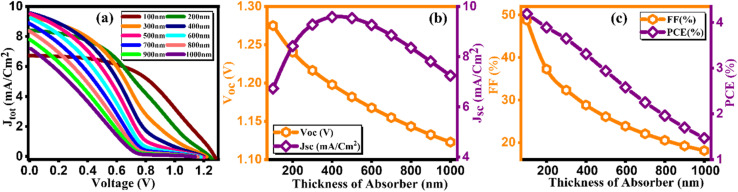
Variation of (a) current density *versus* voltage, (b) *V*_oc_ and *J*_sc_, and (c) FF and PCE with thicknesses of Cs_2_AgBiBr_6_.

Perovskite, serving as an absorber layer, performs a pivotal part in dictating the overall performance of solar cells. Among various parameters influencing the device's functionality, the thickness of the absorber layer stands out as a critical factor.^36^ From Fig. 5(a) we observed the response of current density *versus* voltage plot of the absorber layer at different thicknesses. In our computational analysis, our primary focus revolves around evaluating key device performance metrics such as *J*_sc_, *V*_OC_, FF, and PCE with varying absorber thicknesses, which span the range from 100 to 1000 nm as cited in Table 5.^37^ It's important to note that all other parameters remain constant. As visually represented in Fig. 5(b and c), outcomes of our simulations clearly demonstrate that an increase in absorber thickness leads to a noticeable rise in *J*_sc_, peaking at approximately 9.57 mA cm^−2^ around the 500 nm thickness mark before experiencing a slight decline. In the context of a thin perovskite absorber, the charge carrier's diffusion length surpasses the thickness of the absorber, enabling the majority of excess carriers to efficiently reach their respective electrodes, thereby generating electrical power. The increment in thickness subsequently results in enhanced light absorption and an increased concentration of excess carriers, which in turn elevates *J*_sc_ values.^38^ This phenomenon is primarily attributed to the remarkably high absorption coefficient inherent in perovskite materials, which can often reach up to 10^5^ cm^−1^. Consequently, thin absorber configurations tend to yield considerably high *J*_sc_ and PCE values, as demonstrated in Fig. 5(b and c). The behavior of *V*_oc_ follows a distinct pattern, attaining an optimal value of 1.27 V at 100 nm thickness, after which it exhibits a steep decline, as depicted in Fig. 5(b). *V*_oc_ is defined by equation given as:4
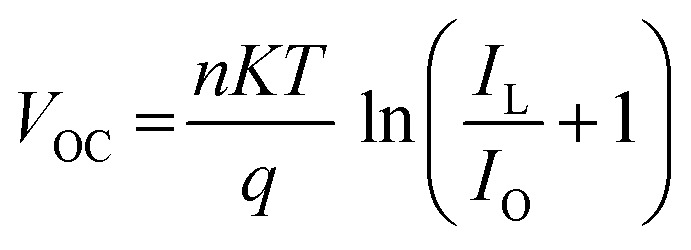


**Table 5:** Variation of performance indicators with thickness of absorber

Thickness (nm)	*V*_OC_ (V)	*J*_SC_ (mA cm^−2^)	FF (%)	PCE (%)
100	1.27	6.72	48.77	4.19
200	1.24	8.41	37.27	3.89
300	1.21	9.27	32.33	3.65
400	1.19	9.57	28.82	3.31
500	1.18	9.53	26.09	2.94
600	1.16	9.26	23.88	2.58
700	1.15	8.85	22.06	2.25
800	1.14	8.35	20.53	1.96
900	1.13	7.80	19.23	1.7
1000	1.12	7.24	18.11	1.47

In the above equation, *n* represents the ideality factor, *KT*/*q* signifies the thermal voltage, *I*_L_ stands for the current generated by light, and *I*_O_ denotes the current under dark saturation conditions. During the stage of increasing *V*_oc_, the recombination of holes and electrons is reduced in thinner absorber layers, maintaining a consistently low level.^39^ This leads to a higher concentration of excess carriers, which in turn facilitates the generation of a large *V*_oc_ and promotes its emergence. However, in the stage of decreasing *V*_oc_, thicker absorber layers elevate to a higher level and provide more opportunities for carrier recombination, consequently leading to a pronounced drop in *V*_oc_. The Fill Factor (FF), as presented in Fig. 5(c), constantly reduces from 48.77 to 18.11 as the thickness of the absorber varies from 100–1000 nm. The FF is a measure of a solar cell's ability to efficiently deliver the generated power to an external load, representing internal power losses. In thicker absorbers, internal power losses intensify, resulting in a reduction of the FF. Fig. 5(c) provides further insights into the performance, particularly power conversion efficiency (PCE), which reaches its peak (4.19%) at 100 nm thickness but exhibits rapid decline with increasing thickness. Our simulations reveal that the efficiency of single junction perovskite solar cells (SJPSCs) is primarily governed by two aspects: carrier transport and photon absorption. Carrier transport becomes the dominant factor in thicker absorber layers, while photon absorption takes precedence in thinner absorber configurations. Consequently, it becomes evident that an optimal absorber thickness, typically ranging from 100 to 400 nm, corresponds to the highest achievable PCE. Beyond this optimal range, excessive absorber thickness leads to an increased presence of excess carriers and traps, creating a higher likelihood of recombination events, thus diminishing overall efficiency.

### 3.3. Effect of electron affinity for hole and electron transport layer

**Fig. 6:**
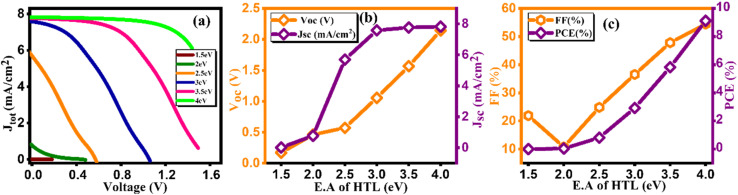
Variation of (a) current density *versus* voltage, (b) *V*_oc_ and *J*_sc_, and (c) FF and PCE with electron affinity of HTL.

**Fig. 7:**
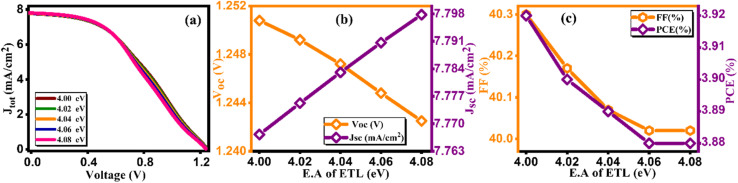
Variation of (a) current density *versus* voltage, (b) *V*_oc_ and *J*_sc_, and (c) FF and PCE with electron affinity of ETL.

At the initial level of simulation, we want to probe the most suitable electron affinity value for HTL through the simulation process. Subsequently, this optimized value was incorporated into physical parameters listed in SCAPS-1D to facilitate additional simulations. The electron affinity is intricately connected with the lowest unoccupied molecular orbital (LUMO) of HTL and ETL and can be described as the energy required (in eV) to elevate free electron from the lowest point of LUMO (or conduction band in case of typical semiconductors) to the vacuum level. Aligning electron affinity with the appropriate energy band gap is crucial for enhancing the performance of the highest occupied molecular orbital (HOMO).^40^ This contributes to improved functioning of the electron/hole injection and blocking processes among perovskite material and HTL/ETL. However, it's essential to establish a practical numerical range for electron affinity values based on reported data for HTL before commencing simulation. This precaution is vital to avoid obtaining unrealistic physical parameters as outputs from the simulation results. Tables 6 and 7 present pertinent electronic parameters, including electron affinity, for the most commonly reported HTL and ETL, respectively. Fig. 6(a) and Fig. 7(a) demonstrate current density *versus* voltage plots for several electron affinity of HTL and ETL, respectively. From our analysis of the data, it's apparent that higher electron affinity is 4 eV for the electron/hole transport layer.^41^ Consequently, the feasible range of electron affinity values for our simulation falls within the bracket of 1.5 to 4.0 eV for HTL and 4 to 4.08 eV for ETL. We proceeded to calculate photovoltaic parameters by systematically varying the electron affinity values. Our objective was to identify the optimum electron affinity for the HTL and ETL in the context of Cs_2_AgBiBr_6_ based solar cell. The outcomes for HTL and ETL, including *V*_oc_, *J*_sc_, FF, and PCE, are presented in Fig. 6(b, c), 7(b and c), respectively. These figures illustrate that the photovoltaic parameters, particularly *V*_oc_, *J*_sc_, and FF, reach their maximum values, resulting in peak PCE of approximately 9.12% for HTL and 3.92% for ETL when electron affinity of HTL and ETL is set at 4.0 eV. Notably, the figure reveals that enhancing the electron affinity of HTL leads to improvements in *V*_oc_, *J*_sc_, FF, and PCE. From Fig. 6(c), the increase in PCE correlates with increased electron affinity of HTL, optimizing electron extraction and minimizing charge recombination at the HTL/active layer interface in solar cells. Enhanced electron affinity aligns energy levels, improving charge transport efficiency and reducing energy losses, resulting in higher observed PCE values. Conversely, the rising electron affinity of ETL is linked to declining PCE due to unfavorable charge transport or increased recombination at the ETL/active layer interface, diminishing overall solar cell efficiency. This investigation unveils the effective role of electron affinity of HTL and ETL in optimizing the overall performance of solar cells.^42^

**Table 6:** Variation of performance indicators with electron affinity of HTL

E.A of HTL (eV)	*V*_OC_ (V)	*J*_SC_ (mA cm^−2^)	FF (%)	PCE (%)
1.5	0.16	0.01	21.89	0
2	0.46	0.77	10.76	0.04
2.5	0.57	5.69	24.82	0.81
3	1.05	7.58	36.53	2.92
3.5	1.56	7.76	47.82	5.82
4	2.14	7.81	54.41	9.12

**Table 7:** Variation of performance indicators with electron affinity of ETL

E.A of ETL (eV)	*V*_OC_ (V)	*J*_SC_ (mA cm^−2^)	FF (%)	PCE (%)
4	1.25	7.76	40.3	3.92
4.02	1.25	7.77	40.17	3.9
4.04	1.25	7.78	40.07	3.89
4.06	1.24	7.79	40.02	3.88
4.08	1.24	7.79	40.02	3.88

### 3.4. Effect of electron affinity of absorber layer

**Fig. 8:**
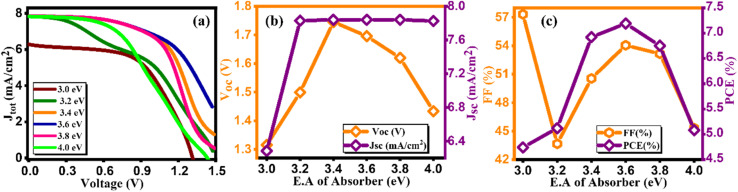
Variation of (a) current density *versus* voltage, (b) *V*_oc_ and *J*_sc_, and (c) FF and PCE with electron affinity of Cs_2_AgBiBr_6_.

Initially, we maintained a constant value of electron affinity for both HTL and ETL, while systematically adjusting the electron affinity of the absorber layer, ranging from 3.0 to 4.0, as depicted in Fig. 8(a–c). Moreover, we plot the current density graph against voltage for different electron affinity of the absorber layer ranging from 3.0–4.0 eV, as illustrated in Fig. 8(a).^43^ The noticeable trend from Fig. 8(b and c) indicates a positive correlation between all four parameters (*V*_OC_, *J*_SC_, FF, and PCE) and a rise in electron affinity as listed in Table 8. As we observed from Fig. 8(c) there is an increase and then subsequent decrease in power conversion efficiency (PCE) with varying electron affinity of the absorber material suggesting an optimal range where charge extraction and carrier transport are most efficient, resulting in higher PCE. Deviations from this optimal range may lead to increased charge recombination or reduced charge extraction, thereby lowering overall efficiency. Remarkably, the simulated device achieved its highest PCE, reaching 7.19%, when electron affinity was set at 3.6 eV, marking a significant milestone in our investigation.^44^

**Table 8:** Variation of performance indicators with electron affinity of absorber

Electron affinity of absorber (eV)	*V*_OC_ (V)	*J*_SC_ (mA cm^−2^)	FF (%)	PCE (%)
3	1.31	6.28	57.32	4.74
3.2	1.49	7.82	43.66	5.12
3.4	1.74	7.84	50.55	6.92
3.6	1.69	7.84	54.05	7.19
3.8	1.61	7.84	53.16	6.75
4	1.43	7.82	45.31	5.08

### 3.5. Effect of defect densities (*N*_t_) of absorber layer

**Fig. 9:**
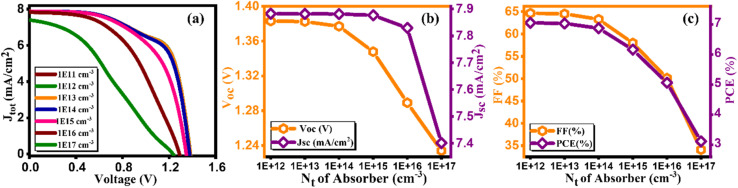
Variation of (a) current density *versus* voltage, (b) *V*_oc_ and *J*_sc_, and (c) FF and PCE with defect density (*N*_t_) of Cs_2_AgBiBr_6_.

The defect density of the absorber layer is one of the crucial parameters that significantly control the efficiency of solar cells. In Cs_2_AgBiBr_6_, encompassing, point defects, interstitial, anti-site, and vacancy types, dominate the defect density landscape, although specific quantification of each type is not provided.^45^ The effectiveness of double perovskite solar cells is notably influenced by the quality and morphology of the perovskite layer. Suboptimal layer characteristic leads to an increase in defect density, resulting in an amplified recombination rate within the absorbing layer, consequently impeding solar cell performance. Fig. 9(a) illustrates the current density *versus* voltage graph for diverse defect densities (*N*_t_) of Cs_2_AgBiBr_6_, spanning from 10^11^–10^17^ cm^−3^. Fig. 9(b and c) showcases how the *V*_oc_, *J*_sc_, FF, and PCE vary in response to defect density (*N*_t_), and their calculated values are mentioned in Table 9.^46^ As depicted in Fig. 9(c), device efficiency initiates at 7.05% at 10^11^, and 10^12^ cm^−3^, gradually decreasing to 7.03% at 10^13^, 6.87% at 10^14^, 6.16% at 10^15^, 5.06% at 10^16^, and 3.11% at 10^17^ cm^−3^. The *V*_oc_, *J*_sc_, and FF values are also plotted in Fig. 9(b and c). The optimized values for these parameters are *V*_oc_ of 1.38 V, *J*_sc_ of 7.88 mA cm^−2^, FF of 64.66%, and an efficiency of 7.05% achieved at a defect density of 10^14^ cm^−3^. The reduction in PV parameters with increasing defect density is attributed to the impact of defects on charge carrier recombination and transport. At lower defect densities, the device efficiency is relatively high due to fewer defects hindering charge movement. As the defect density increases, more defects act as non-radiative recombination centers, reducing the open-circuit voltage (*V*_oc_), short-circuit current density (*J*_sc_), and fill factor (FF), ultimately leading to decreased device efficiency.

**Table 9:** Variation of performance indicators with defect density of absorber

Defect density of absorber (cm^−3^)	*V*_OC_ (V)	*J*_SC_ (mA cm^−2^)	FF (%)	PCE (%)
1 × 10^11^	1.38	7.88	64.66	7.05
1 × 10^12^	1.38	7.88	64.65	7.05
1 × 10^13^	1.38	7.88	64.51	7.03
1 × 10^14^	1.37	7.88	63.29	6.87
1 × 10^15^	1.34	7.87	57.98	6.16
1 × 10^16^	1.28	7.82	50.12	5.06
1 × 10^17^	1.23	7.40	34.08	3.11

### 3.6. Effect of doping concentration (*N*_A_) of absorber layer

**Fig. 10:**
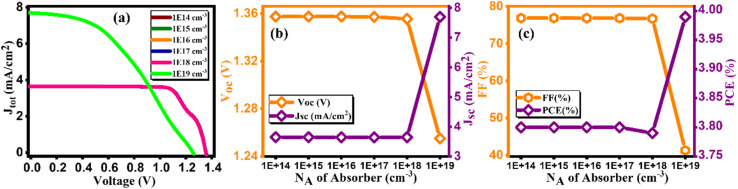
Variation of (a) current density *versus* voltage, (b) *V*_oc_ and *J*_sc_, and (c) FF and PCE with doping concentration (*N*_A_) of Cs_2_AgBiBr_6_.

Fig. 10(a) provides a visual representation of current density *versus* voltage characteristics for varying the doping concentration in Cs_2_AgBiBr_6_, spanning from 10^14^ to 10^19^ cm^−3^. Intending to investigate the impact of varying doping concentrations of Cs_2_AgBiBr_6_ on device efficiency, we conducted a simulation study within the range of 10^14^ to 10^19^ cm^−3^.^47^ Observations from Fig. 10(b and c) reveal an initial increase in *J*_SC_, FF, and overall performance up to a particular threshold. However, further increments in doping content result in a decline in the output of a device, accompanied by a reduction in *V*_OC_ from 1.35 to 1.25 V. The device achieved its highest PCE of 3.99% at 10^19^ cm^−3^ as tabulated in Table 10. Notably, there exists a correlation between doping concentration and electric field; as doping content rises, the electric field strength also rises. This upsurge in the electric field promotes rapid charge carrier separation, consequently enhancing device efficiency. In its optimized state, the device exhibited a *V*_OC_ of 1.25 V, *J*_SC_ of 7.69 mA cm^−2^, FF of 41.31%, and PCE of 3.99%.^48^

**Table 10:** Variation of performance indicators with doping concentration of absorber

Doping concentration *N*_A_ of absorber	*V*_OC_ (V)	*J*_SC_ (mA cm^−2^)	FF (%)	PCE (%)
1 × 10^14^	1.35	3.64	76.84	3.80
1 × 10^15^	1.35	3.64	76.84	3.80
1 × 10^16^	1.35	3.64	76.83	3.80
1 × 10^17^	1.36	3.64	76.82	3.80
1 × 10^18^	1.36	3.64	76.7	3.79
1 × 10^19^	1.25	7.69	41.31	3.99

### 3.7. Effect of doping concentration (*N*_A_) of HTL

**Fig. 11:**
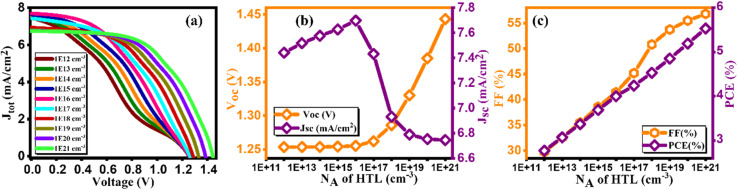
Variation of (a) Current density *versus* voltage, (b) *V*_oc_ and *J*_sc_, and (c) FF and PCE with doping concentration (*N*_A_) of HTL.

Fig. 11(a) illustrates the current density/voltage characteristics for a range of Cu_2_O doping contents, varying from 10^12^ to 10^21^ cm^−3^. The figure indicates that higher concentrations result in improved efficiency, attributed to enhanced extraction and charge transport at the Cu_2_O/absorber interface. In Fig. 11(b and c), the impact of acceptor doping concentration (*N*_A_) on performance parameters is depicted.^49^ It is observed that PCE is lower at lower *N*_A_ levels, primarily due to elevated series resistance, aligning with prior research findings. Consequently, as demonstrated in Fig. 11(c), PCE is maximized at 10^21^ cm^−3^. This configuration yields *V*_oc_ of 1.44 V, Jsc of 6.74 mA cm^−2^, FF of 56.8%, and an impressive efficiency of 5.53% as listed in Table 11.^50^

**Table 11:** Variation of performance indicators with doping concentration of HTL

Doping concentration *N*_A_ of HTL	*V*_OC_ (V)	*J*_SC_ (mA cm^−2^)	FF (%)	PCE (%)
1 × 10^12^	1.25	7.44	29.72	2.77
1 × 10^13^	1.25	7.51	32.56	3.07
1 × 10^14^	1.25	7.57	35.49	3.37
1 × 10^15^	1.25	7.62	38.56	3.69
1 × 10^16^	1.25	7.69	41.41	4.00
1 × 10^17^	1.26	7.43	45.18	4.24
1 × 10^18^	1.28	6.93	50.8	4.53
1 × 10^19^	1.33	6.78	53.73	4.85
1 × 10^20^	1.38	6.75	55.47	5.19
1 × 10^21^	1.44	6.74	56.8	5.53

### 3.8. Effect of doping concentration (*N*_D_) of ETL

**Fig. 12:**
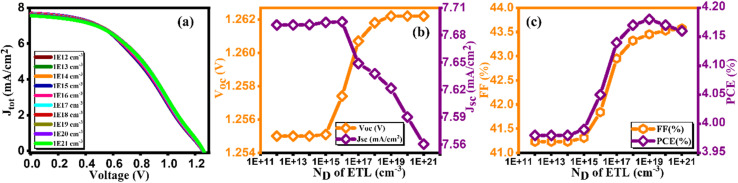
Variation of (a) current density *versus* voltage, (b) *V*_oc_ and *J*_sc_, and (c) FF and PCE with doping concentration (*N*_D_) of ETL.

Fig. 12(a) exhibits a current density *versus* voltage graph for varying doping contents of GO, ranging from 10^12^ to 10^21^ cm^−3^. It is evident from this figure that higher concentrations result in enhanced extraction and charge transport at the GO/absorber interface. In Fig. 12(b and c), the impact of donor doping concentration (*N*_D_) on performance parameters is depicted. Once again, it is evident that PCE is adversely affected at lower *N*_D_ levels, primarily due to increased series resistance, which aligns with the findings of previous studies.^51^ Consequently, as illustrated in Fig. 12(c), PCE reaches its maximum value of 4.18% at 10^19^ cm^−3^. This configuration yields *V*_oc_ of 1.26 V, *J*_sc_ of 7.56 mA cm^−2^, and FF of 43.57% as mentioned in Table 12, representing notable achievement in solar cell efficiency.^52^

**Table 12:** Variation of performance indicators with doping concentration of ETL

Doping concentration *N*_D_ of ETL	*V*_OC_ (V)	*J*_SC_ (mA cm^−2^)	FF (%)	PCE (%)
1 × 10^12^	1.25	7.69	41.23	3.98
1 × 10^13^	1.25	7.69	41.23	3.98
1 × 10^14^	1.25	7.69	41.23	3.98
1 × 10^15^	1.25	7.69	41.31	3.99
1 × 10^16^	1.25	7.69	41.84	4.05
1 × 10^17^	1.26	7.64	42.95	4.14
1 × 10^18^	1.26	7.63	43.32	4.17
1 × 10^19^	1.26	7.62	43.45	4.18
1 × 10^20^	1.26	7.59	43.53	4.17
1 × 10^21^	1.26	7.56	43.57	4.16

### 3.9. Effect of temperature on the cell performance

**Fig. 13:**
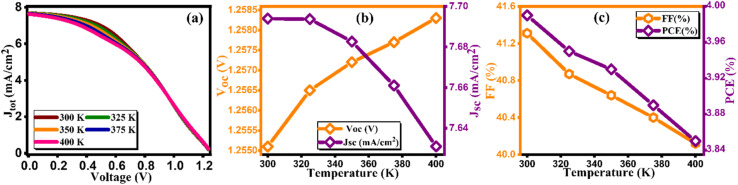
Variation of (a) current density *versus* voltage, (b) *V*_oc_ and *J*_sc_, and (c) FF and PCE with temperature.

Perovskite solar cells (PSCs) offer simplicity in manufacturing and high light absorption efficiency, but their widespread adoption is hindered by stability issues. Factors like temperature, humidity, moisture, and ultraviolet radiation have notable impacts on PSC performance. Temperature, in particular, plays a critical role, as photovoltaic devices often operate under direct sunlight, which can lead to temperatures up to 45 °C higher than the ambient conditions.^53^ Temperature variations influence the semiconductor properties; lower temperatures narrow the semiconductor's bandgap, while higher temperatures increase the atom spacing and improve conductivity and electrical mobility. Fig. 13(a) illustrates the current density *versus* voltage plot. However, there is a noticeable correlation between elevated temperatures and increment in *V*_oc_ as demonstrated in Fig. 13(b). This correlation can be attributed to the destabilization of electrons at high temperatures, increasing the likelihood of electron–hole recombination. The increased temperature also introduces more frequent scattering events, primarily lattice scattering, which hinders electron and hole movement. Consequently, carrier movement decreases due to scattering at higher temperatures, resulting in increased cell resistance. This effect is reflected in the decrease in fill factor (FF) from 41.31% to 40.12% in Fig. 13(c). Overall, the device's performance diminishes, leading to a reduction in PCE from 3.99% to 3.85% as cited in Table 13.^54^ This reduction in PCE with increasing temperature indicates thermal degradation of the solar cell components, leading to reduced performance due to increased carrier recombination and altered material properties at higher temperatures.

**Table 13:** Variation of performance indicators with environmental temperature

Temperature (K)	*V*_OC_ (V)	*J*_SC_ (mA cm^−2^)	FF (%)	PCE (%)
300	1.25	7.69	41.31	3.99
325	1.25	7.69	40.87	3.95
350	1.25	7.68	40.64	3.93
375	1.25	7.66	40.4	3.89
400	1.25	7.63	40.12	3.85

### 3.10. Effect of change in series resistance

**Fig. 14:**
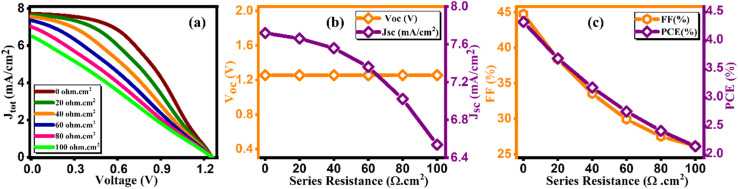
Variation of (a) current density *versus* voltage, (b) *V*_oc_ and *J*_sc_, and (c) FF and PCE with series resistance.

In studies conducted under intense light, it has been established that the device's efficiency experiences a marginal improvement as a result of increasing light intensity. Nevertheless, it is imperative to note that the presence of series resistance (*R*_s_) leads to certain inefficiencies within the PSC.^55^ Supplementary material illustrates the performance of the PSC device, wherein series resistance changes within the range of 0–100 Ω cm^2^ as depicted in Fig. 14(a–c). The current density *versus* voltage graph is plotted in Fig. 14(a). Notably, while series resistance exerts a limited influence on *V*_OC_, it exerts a substantial impact on both *J*_SC_ and FF. This, in turn, culminates in a reduction of overall efficiency in perovskite solar cells. The interplay among *V*_OC_ and *J*_SC_ within PSCs might be effectively elucidated in the application of the diode equivalent circuit model, as mathematically expressed through the ensuing equations:^56^5
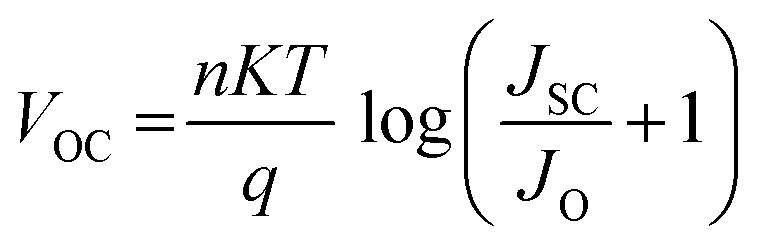
6
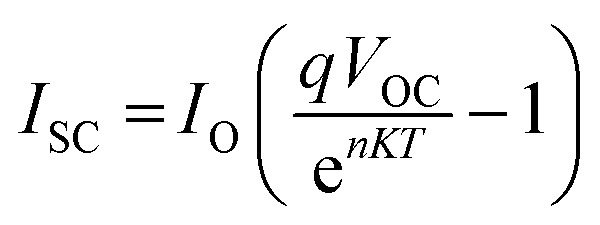
7
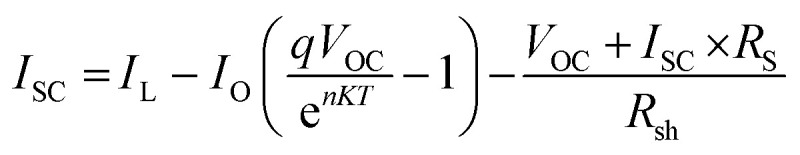


In this context, the term *V*_OC_ pertains to voltage at an open circuit, while *I*_L_ signifies current generated by incident light. *I*_sc_ represents current at short circuit, and *R*_S_ and *R*_sh_ denote resistances in series and shunt, respectively. *J*_SC_ stands for current density, *q* signifies reverse saturation current, *n* represents ideality factor, *K* is Boltzmann constant, and *T* indicates temperature. Upon a close examination of the aforementioned equations, it becomes evident that an escalation in *R*_S_ leads to a notable reduction in *I*_sc_, thereby directly impacting both FF and PCE. Within our current investigation, *R*_S_ is subjected to variation within the range of 0 to 100 Ω cm^2^, which results in a decrease in the value of *J*_SC_ (ranging from 7.72 to 6.54 mA cm^−2^) as illustrated in Fig. 14(b). This reduction in *J*_SC_, in turn, leads to a decline in FF (44.75%–26.15%) and device efficiency (4.33%–2.15%) as shown in Fig. 14(c) and cited in Table 14. It is noteworthy that *V*_OC_ remains almost constant throughout this simulation. This constant value of *V*_OC_ despite increasing series resistance suggests that series resistance does not significantly impact *V*_OC_ in this scenario, indicating a possible dominance of other factors (such as shunt resistance or intrinsic material properties) in determining *V*_OC_ in the solar cell. Furthermore, under ideal conditions, specifically at 0 Ω cm^2^, the simulated device showcased its highest attainable PCE, reaching 4.33%, as visually represented in Fig. 14(c). Overall, the decrease in PCE with increasing *R*_S_ indicates that higher resistance leads to increased voltage losses and reduced current output, impacting overall efficiency by limiting charge transport and increasing energy dissipation within the solar cell.

**Table 14:** Variation of performance indicators with series resistance

Series resistance (ohm cm^2^)	*V*_OC_ (V)	*J*_SC_ (mA cm^−2^)	FF (%)	PCE (%)
0	1.25	7.71	44.75	4.33
20	1.25	7.66	38.36	3.69
40	1.25	7.55	33.53	3.18
60	1.25	7.36	29.91	2.76
80	1.25	7.02	27.49	2.42
100	1.25	6.53	26.15	2.15

### 3.11. QE characteristics

**Fig. 15:**
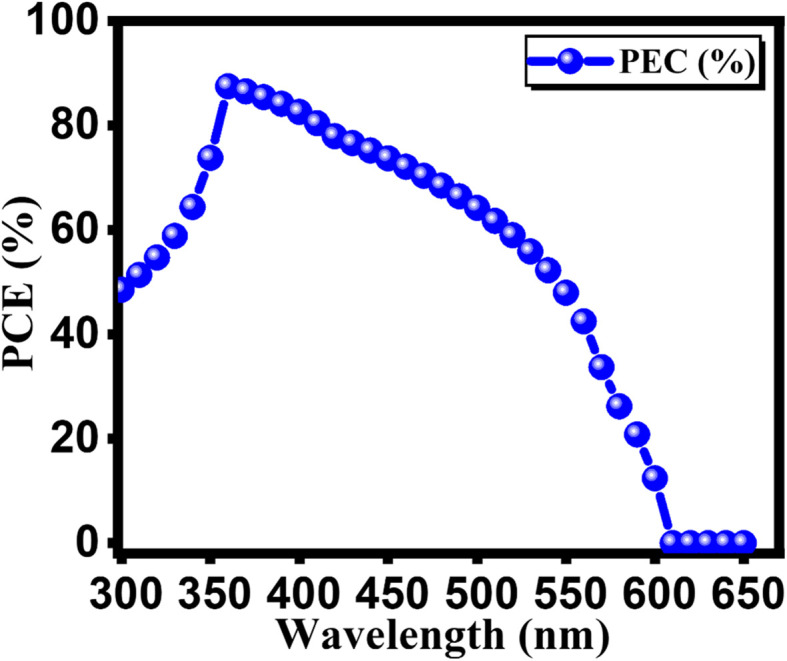
Calculated quantum efficiency for FTO/GO/Cs_2_AgBiBr_6_/Cu_2_O.

In Fig. 15, we observe the quantum efficiency (QE) variations with respect to the wavelength for double perovskite solar cell structure, both in the initial and final stages of optimization. The wavelength of incident light plays a crucial role in influencing QE.^57^ This parameter represents the ratio of charge carriers produced by solar cells to the incident photons striking semiconductor material. This relationship is well-documented in the literature.^58^ Additionally, it's worth noting that a thicker absorber tends to enhance photon absorption, resulting in an overall improvement in QE. This trend is supported by previous studies. Interestingly, our observations indicate that QE has the highest value at a wavelength of 350 nm, regardless of whether we consider the initial or final optimized conditions. However, beyond this range, there is a noticeable decrease in QE, confirming the intricate interplay between incident light wavelength and quantum efficiency. This finding holds true for both the initial and final optimization stages, as illustrated in Fig. 15.^59^

### 3.12. Results of SCAPS-1D compared to previous research

In Table 15, we conduct a comprehensive comparison of photovoltaic performance parameters, taking into account recently published theoretical research work alongside our study. The data presented in the table unequivocally demonstrates that our work has achieved a notably higher Power Conversion Efficiency (PCE) in comparison to previously reported device configurations utilizing Cs_2_AgBiBr_6_.

**Table 15:** Comparison of PV parameters of Cs_2_AgBiBr_6_-based solar cells with the present study

Device structure	*V*_OC_ (V)	*J*_SC_ (mA cm^−2^)	FF (%)	PCE (%)	Ref.
ITO/SnO_2_/Cs_2_AgBiBr_6_/P3HT/Au	1.09	1.73	0.76	1.44	60
ITO/SnO_2_/Cs_2_AgBiBr_6_/Cu_2_O/Au	1.17	3.42	0.78	3.13	60
FTO/cTiO_2_/mTiO_2_/Cs_2_AgBiBr_6_/N719/spiro-OMeTAD/Ag	1.06	5.13	—	2.84	61
FTO/TiO_2_/Cs_2_AgBiBr_6_/Sipro-OMeTAD/Au	0.98	3.96	62.4	2.43	62
FTO/GO/Cs_2_AgBiBr_6_/Cu_2_O	2.14	7.81	54.41	9.12	Our work

## 4. Conclusion

In our research, we conducted an extensive simulation using the SCAPS software, focusing on the exploration of inorganic, Pb-free Cs_2_AgBiBr_6_ perovskites for potential photovoltaic device applications. Our study examined various parameters, including the thickness of the electron transport layer (ETL), hole transport layer (HTL), and absorber, as well as defect density, doping concentration, electron affinity, temperature, and series resistance. Notably, the optimized results, for the Cs_2_AgBiBr_6_ perovskite device, underscored significant impacts stemming from variations in series resistance and temperature. Furthermore, the present study demonstrates a significant improvement in the PEC (9.12%) compared to the previously reported PEC values of 1.44, 3.13, 2.84, and 2.43% of double perovskite-based solar cells. Collectively, our findings offer not only encouragement but also valuable insights, paving the way toward the realization of inorganic and nontoxic perovskite solar cell devices. This research represents a substantial advancement in addressing environmental concerns associated with perovskite technology.

## Data availability

All data included in this work can be provided on reasonable request.

## Author contributions

Ghulam M. Mustafa: conceptualization, writing original draft, Bisma Younas: software, writing original draft, investigation, Sadaf Saba: writing original draft, methodology, software, Zainab Mufarreh Elqahtani: investigation; funding acquisition; software; resources, Norah Alwadai: project administration; formal analysis; writing – review & editing, Sikandar Aftab: resources and supervision

## Conflicts of interest

There are no conflicts to declare.

## Supplementary Material
